# Early Brain Metastasis despite Pathological Complete Response after Radical Resection for Esophageal Cancer following Chemotherapy and Pembrolizumab

**DOI:** 10.70352/scrj.cr.25-0455

**Published:** 2025-09-19

**Authors:** Tetsuhiro Urashima, Yusaku Tanaka, Kota Imanishi, Sachika Kinoshita, Shogo Takei, Yasuhiro Shimizu, Taichi Yabuno, Yasuhisa Mochizuki

**Affiliations:** Department of Gastrointestinal Surgery, Yokohama Municipal Citizen’s Hospital, Yokohama, Kanagawa, Japan

**Keywords:** brain metastasis, conversion surgery, esophageal cancer, pathological complete response, pembrolizumab

## Abstract

**INTRODUCTION:**

Brain metastasis from esophageal cancer is rare. In particular, recurrent brain metastasis following multimodal treatment, such as preoperative chemotherapy and surgical resection, is extremely uncommon.

We present a case of early brain metastasis from esophageal cancer despite achieving pathological complete response (pCR).

**CASE PRESENTATION:**

A middle-aged man presented with dysphagia. Upper gastrointestinal endoscopy revealed stenosis caused by an ulceroinfiltrative tumor located in the middle of the thoracic esophagus. Targeted biopsies confirmed squamous cell carcinoma. Contrast-enhanced CT revealed circumferential, irregular wall thickening with contrast enhancement, showing infiltration into the left main bronchus and enlarged lymph nodes in the paraesophageal and the left supraclavicular regions. The diagnosis was middle thoracic esophageal squamous cell carcinoma cT3br N2M0 cStage IIIB (according to the Japanese Classification of Esophageal Cancer, 12th Edition). The patient underwent chemotherapy, including 5-fluorouracil, cisplatin, and pembrolizumab, as the combined positive score exceeded 10. Following this chemotherapy with an immune checkpoint inhibitor, the tumor had regressed, and targeted biopsies revealed no malignant findings. The post-chemotherapy diagnosis was ycT3rN0M0 ycStage II, and the patient subsequently underwent thoracoscopic esophagectomy. Surgical findings showed no evidence of tumor infiltration. Postoperative histopathological examination showed no residual tumor cells in either the esophagus or resected lymph nodes, corresponding to histological response of grade 3. However, the patient presented with depression 2 months after the surgery, and abnormal behavior was shown 3 months after the surgery. Although cranial CT and MRI revealed ring-enhancing lesions in the right cerebellar hemisphere and the right frontal lobe, there was no recurrence or metastasis other than in the brain. The patient underwent resection of the frontal lobe tumor and was diagnosed with brain metastasis of esophageal cancer. Stereotactic radiation therapy and pembrolizumab were started; however, the patient died 5 months after esophagectomy due to brain metastasis progression.

**CONCLUSIONS:**

5-Fluorouracil and cisplatin plus pembrolizumab therapy may allow conversion surgery in advanced esophageal cancer. However, even in patients who achieve pCR at the primary lesion, brain metastasis may occur after surgical treatment. Preoperative and postoperative surveillance for brain metastases is necessary in patients at high risk of distant metastasis, even if the local lesion is controlled.

## Abbreviations

BBB
: blood–brain barrier
CPS
: combined positive score
FP
: 5-fluorouracil and cisplatin
iBMEC
: isolated brain metastasis from esophageal carcinoma
ICI
: immune checkpoint inhibitor
pCR
: pathological complete response
sBMEC
: systemic brain metastasis from esophageal carcinoma

## INTRODUCTION

The common distant metastases of esophageal cancer include the lungs, liver, bones, and lymph nodes, while brain metastases are rare. In particular, recurrent brain metastasis following multimodal treatment such as preoperative chemotherapy and surgical resection is extremely uncommon.

We report a case in which pathological complete response (pCR) was achieved in both the primary esophageal cancer and the metastatic lymph nodes following chemotherapy. However, an isolated brain metastasis from esophageal carcinoma (iBMEC) developed during the early postoperative period.

## CASE PRESENTATION

**Fig. 1:**
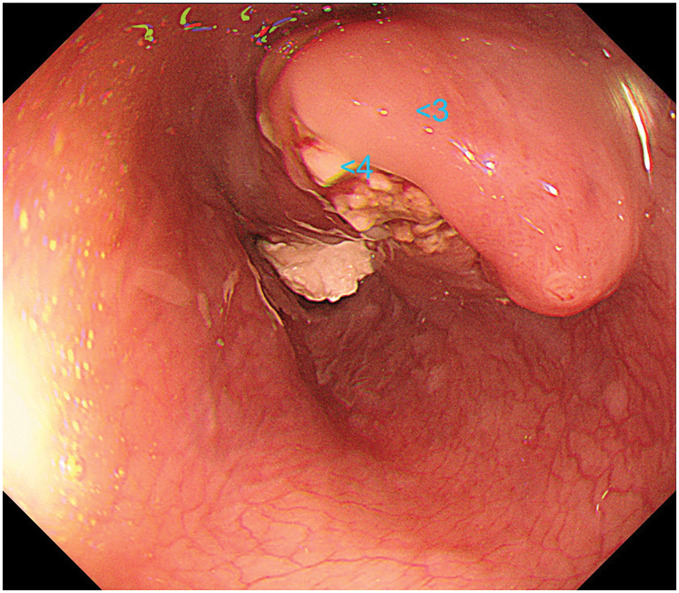
Upper gastrointestinal endoscopy before FP plus pembrolizumab. A stenotic ulcerative infiltrative tumor in the middle of the thoracic esophagus, and a targeted biopsy revealed squamous cell carcinoma (blue arrow). FP, 5-fluorouracil and cisplatin

**Fig. 2:**
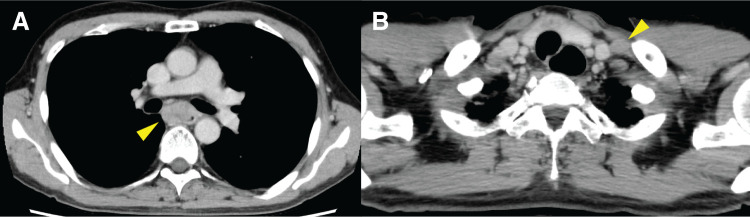
Contrast-enhanced CT before FP plus pembrolizumab. (**A**) Irregular wall thickening with contrast enhancement, suggesting infiltration into the left main bronchus (yellow arrow). (**B**) Enlarged lymph nodes in the paraesophageal and the left supraclavicular areas (yellow arrow).

A middle-aged man with no significant past medical history other than hypertension presented with dysphagia. Upper gastrointestinal endoscopy revealed stenosis caused by an ulcerative infiltrative tumor in the middle of the thoracic esophagus, located between 30 and 37 cm from the incisors (**Fig. 1**). Targeted biopsies revealed squamous cell carcinoma. Contrast-enhanced CT revealed circumferential, irregular wall thickening with contrast enhancement extending over 5.5 cm in the middle of the thoracic esophagus (**Fig. 2A**). Infiltration into the left main bronchus was suspected due to an unclear membrane structure between the tumor and the left main bronchus. In addition, CT also revealed enlarged lymph nodes in the paraesophageal and the left supraclavicular areas (**Fig. 2B**), but no other distant metastases were detected. Preoperative cranial CT imaging was not performed. The diagnosis before the treatment was middle thoracic esophageal squamous cell carcinoma cT3br (suspicion of infiltration into the left bronchus) N2M0 cStage IIIB (according to the Japanese Classification of Esophageal Cancer, 12th Edition).

**Fig. 3:**
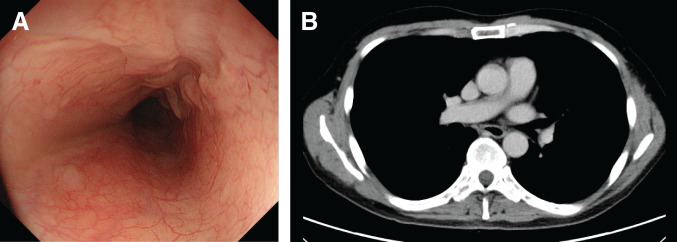
Upper gastrointestinal endoscopy and contrast-enhanced CT after FP plus pembrolizumab. (**A**) The tumor lesion had flattened and was covered with normal mucosa. (**B**) The tumor and enlarged lymph nodes had reduced in size. FP, 5-fluorouracil and cisplatin

**Fig. 4:**
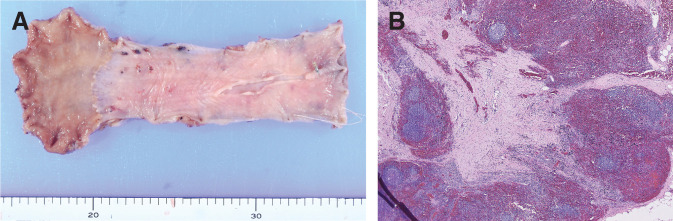
Pathological specimen. (**A**) Postoperative macroscopic findings revealed fibrosis of the esophageal mucosa. (**B**) No residual tumor cells were found in the esophagus or the resected lymph nodes.

Chemotherapy was suggested because radical resection was difficult, and chemoradiotherapy carries a higher risk of complications than chemotherapy alone. The chemotherapy regimen included 5-fluorouracil and cisplatin (FP) plus pembrolizumab, as the combined positive score was higher than 10. The dose of cisplatin was reduced to 75% from the 2nd course onwards due to Grade 2 neutropenia and Grade 1 elevation of serum creatinine as adverse events during the administration period. Upper gastrointestinal endoscopy performed after the 4th course of FP plus pembrolizumab showed that the tumor lesion had flattened and was covered with normal mucosa (**Fig. 3A**). Targeted biopsies revealed no malignant findings. Contrast-enhanced CT showed that the tumor and enlarged lymph nodes had reduced in size (**Fig. 3B**). The diagnosis after chemotherapy was ycT3rN0M0 ycStage II, and the patient underwent thoracoscopic esophagectomy, 3-field lymph node dissection, and gastric tube reconstruction via the retrosternal route. In the surgical findings, the adhesion between the esophagus and the left main bronchus was exhibited; however, there was no infiltration, and the dissection was enabled. Postoperative macroscopic findings revealed fibrosis of the esophageal mucosa (**Fig. 4A**), and histopathological findings showed fibrosis extending from the lamina propria to the submucosal layer (**Fig. 4B**). No residual tumor cells were found in the esophagus or the resected lymph nodes. The postoperative diagnosis was pCR, and the histological response was Grade 3. Although anastomotic leakage was present, the patient was treated conservatively and discharged 45 days after the surgery.

**Fig. 5:**
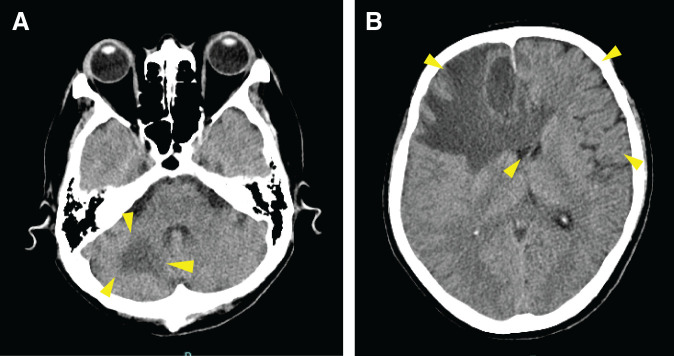
Postoperative cranial CT. (**A**) A ring enhancement in the right cerebellar hemisphere (yellow arrows). (**B**) Ring enhancement was also detected in the right frontal lobe (yellow arrows).

Two months after the surgery, the patient presented with depression and loss of appetite. Imaging did not reveal any clear findings of gastrointestinal obstruction. Three months after the surgery, the patient exhibited abnormal behavior, and cranial CT and MRI revealed ring enhancement in the right cerebellar hemisphere and right frontal lobe (**Fig. 5A** and **5B**). Despite the systemic work-up, there was no recurrence or metastasis other than in the brain. Craniotomy was performed to resect a frontal lobe tumor for diagnostic purposes due to suspicion of sporadic brain metastasis of esophageal cancer. Postoperative pathological findings revealed squamous cell carcinoma, and a diagnosis of brain metastasis of esophageal cancer was made. The patient was able to eat solid food again after the surgery. Stereotaxic radiation therapy for the brain metastasis was started 7 days after the craniotomy. In addition, 5 courses of pembrolizumab were administered from 8 weeks after the craniotomy; however, the size of the brain metastasis increased. Despite there being no other sites of recurrence or metastasis, the patient died from progression of the brain metastasis 5 months after esophagectomy.

## DISCUSSION

In Japan, the standard treatment for cStage III thoracic esophageal cancer is preoperative chemotherapy with docetaxel, cisplatin, and 5-fluorouracil, followed by surgical resection.^1)^ Treatment options for unresectable advanced esophageal cancer include chemoradiotherapy, chemotherapy alone, and radiotherapy alone. For the primary treatment of unresectable advanced or recurrent esophageal cancer, combining chemotherapy with immune checkpoint inhibitors (ICIs) has been reported to significantly improve overall survival (OS), progression-free survival, and the overall response rate compared to conventional chemotherapy alone.^2)^ Additionally, chemotherapy combined with ICIs has been reported to improve the pCR rate following neoadjuvant chemotherapy in patients who have not undergone radiotherapy.^3)^ As a first-line treatment, either FP plus ICIs (a combination of cytotoxic chemotherapy and ICIs) or dual ICI therapy is recommended.^1)^ In esophageal cancer cases with CPS of 10 or higher, pembrolizumab in combination with chemotherapy has been suggested to be more beneficial than chemotherapy alone.^2)^ However, the standard treatment in European countries consists of preoperative chemoradiotherapy followed by surgical resection. While preoperative chemoradiotherapy offers superior local control compared to preoperative chemotherapy alone, it is also associated with an increased risk of perioperative complications and surgery-related mortality. Preoperative CT imaging suggested bronchial invasion by the tumor; therefore, the treatment strategy was based on protocols for unresectable esophageal cancer. Given that the CPS was 10 or higher, FP plus pembrolizumab therapy was selected. Preoperative radiotherapy was not administered due to concerns regarding perioperative complications and surgery-related mortality.

Brain metastasis is one of the most common complications related to solid tumors, representing a major cause of neurological complications and mortality in cancer patients.^4)^ Esophageal cancer often recurs after radical resection; however, the incidence of brain metastasis from esophageal cancer is relatively low, compared to lung or breast cancer, with reported rates ranging from 0% to 5%.^5,6)^ It has been reported that brain metastases larger than 3 cm account for 53% of cases, and multiple metastases are observed in 52%–64% of patients with brain metastasis from esophageal cancer.^7,8)^ In our case, brain metastases larger than 3 cm were observed.

Brain metastasis following resection of esophageal cancer is classified into 2 patterns: systemic brain metastasis from esophageal carcinoma (sBMEC) and iBMEC. It has been reported that iBMEC is generally associated with a more favorable prognosis compared to sBMEC.^9)^ According to a prospective cohort study involving 1760 cases of esophageal cancer following radical resection, iBMEC was identified in 38 cases (2.3%).^10)^ Due to the blood–brain barrier (BBB), drug permeability in the brain is poor, which allows brain metastases to persist. In cases that respond well to chemotherapy, iBMEC is often observed.^11)^

Brain metastasis from esophageal cancer is associated with a poor prognosis, and the median survival time for iBMEC has been reported as 0.95 years (95% confidence interval: 0.6–1.5).^10)^ The median OS was 0.9 years for recurrent cases, 1.4 years for local recurrence, 1.0 years for iBMEC, and 0.8 years for distant metastasis. More than half of patients were diagnosed within 1 year after the surgery. Furthermore, among iBMEC cases, patients who achieved pCR following preoperative chemotherapy had a significantly longer OS compared to those who did not (median OS: 1.56 years vs 0.66 years, p = 0.019). In our case, the presence of brain metastasis was unknown because cranial imaging was not performed prior to chemotherapy and surgery. Therefore, it cannot be ruled out that brain metastasis was already present before treatment. However, distant metastasis was not detected on systemic CT, except in the brain, due to FP plus pembrolizumab. Moreover, residual tumor cells were not detected in the surgically resected primary lesion and dissected lymph nodes, and pCR was achieved. Monoclonal antibody drugs are known to have large molecular weights that prevent them from crossing the BBB. Thus, in our case as well, the ICI did not act effectively on the intracranial lesions, although it was effective throughout the rest of the body, suggesting the development of isolated brain metastasis. On the other hand, the incidence of brain metastasis within 1 year after esophagectomy was reported to be 6 times higher in patients who underwent postoperative chemotherapy than in those who did not receive chemotherapy.^12)^ In addition, patients who received both preoperative and postoperative chemotherapy had a 9-fold higher incidence.^12)^ While the mechanism is unclear, chemotherapy itself has been suggested as a risk factor for brain metastasis after surgery for esophageal cancer. A PubMed search conducted in December 2024 using the keywords “esophageal cancer,” “brain metastasis,” and “pembrolizumab” found no case reports of brain metastasis occurring in patients who underwent FP plus ICI therapy and achieved pCR. Therefore, this is considered the first reported case.

Risk factors for brain metastasis in esophageal cancer include the following: (1) large tumor size, (2) invasion of the adventitia, (3) lymph node metastasis, and (4) administration of neoadjuvant or adjuvant chemotherapy.^12,13)^ The recurrence rate of distant metastasis after achieving pCR has been reported to be 3% for cT1/2 and 14% for cT3/4, suggesting that patients with deeper tumor invasion are more likely to develop distant metastases.^14)^ In addition, all cases of iBMEC have been reported to have received neoadjuvant chemotherapy.^10)^ A study of 911 patients who underwent esophagectomy following neoadjuvant chemotherapy found that those with residual lesions have a lower frequency of brain metastasis than those diagnosed with pCR (2% vs 4.6%, p = 0.051).^15)^ In our case, bronchial invasion was suspected. The risk of distant metastasis was elevated due to the depth of tumor invasion and preoperative chemotherapy.

There is no established consensus regarding surveillance for brain metastasis following esophageal cancer resection. Although such surveillance is generally considered cost-ineffective,^13,16)^ the frequency of brain metastasis detection has increased in recent years due to advancements in imaging technologies. Some previous reports have suggested that surveillance may be beneficial for patients with high-risk factors.^17)^ In our case, cranial imaging was performed for the 1st time after the appearance of postoperative symptoms such as depression and abnormal behavior, which led to the detection of iBMEC. Preoperative and postoperative cranial imaging is seldom performed because brain metastasis from esophageal cancer is rare, with a reported incidence of 3.8%.^12)^ Most cases of iBMEC were initially detected by CT scans.^10)^ However, in this case, it cannot be ruled out that brain metastasis may have been present prior to the treatment since no cranial imaging was performed before the chemotherapy and the surgery. A previous study reported that locally advanced esophageal cancer has a higher frequency of iBMEC compared to nonlocally advanced cancer (2.87% vs 0.56%). Furthermore, cases with lymph node metastasis have also been reported to have a higher incidence of iBMEC. In addition, approximately 34% of iBMEC appeared within 6 months postoperatively.^10)^ For high-risk cases of distant metastasis, we believe preoperative cranial imaging is necessary, as well as follow-up with cranial imaging within at least 6 months postoperatively. In cases with high risk factors of brain metastasis of esophageal cancer, as iBMEC may still develop early after surgery, surveillance using CT should be considered even if pCR is achieved with chemotherapy.

According to Japanese guidelines,^18)^ treatment for metastatic brain tumors varies depending on the number of tumor lesions. For multiple lesions, whole-brain irradiation is the primary recommendation. Other therapies include stereotactic radiotherapy, pharmacological treatment, and tumor resection. In our case, surgical resection was chosen to differentiate between an infectious brain abscess and a tumor, and to improve symptoms early on by removing the frontal lobe lesion. Therefore, diagnostic surgery was performed first, followed by stereotactic radiotherapy after pathological confirmation of brain metastasis. Additionally, drug therapy is generally considered less effective for brain metastasis due to the BBB. However, some studies suggest that disruption of the BBB caused by tumor progression may allow medications to reach brain lesions and exert therapeutic effects.^19)^ There have been reports of cases in which chemotherapy with ICI was initially ineffective, but subsequent ICI therapy led to favorable responses following surgical resection or radiotherapy of brain tumors.^20)^ Thus, ICI therapy was initiated following resection of the brain metastasis and radiotherapy, considering the systemic condition. The average survival time and 1-year survival rate for patients with a single brain metastasis who underwent tumor resection combined with whole-brain irradiation were 9.6 months and 42%.^8,21)^ The prognosis of patients with brain metastasis from esophageal cancer is poor; however, postoperative follow-up for esophageal cancer should take this condition into account, as identifying brain metastasis as a single lesion and performing surgical resection combined with radiotherapy may improve the prognosis.

## CONCLUSIONS

FP plus pembrolizumab therapy may allow conversion surgery for advanced esophageal cancer. However, iBMEC can still occur even in patients who achieve pCR at the primary lesion. Although brain metastasis can be controlled in a localized lesion, early detection may improve the prognosis. Therefore, preoperative and postoperative surveillance with awareness of the possibility of brain metastasis is necessary in patients at high risk for distant metastasis.

## DECLARATIONS

### Availability of data and materials

The datasets supporting the conclusions of this article are included within the article.
